# Utilizing Optical Coherence Tomography in the Nondestructive and Noncontact Measurement of Egg Shell Thickness

**DOI:** 10.1155/2014/205191

**Published:** 2014-07-13

**Authors:** Metin Sabuncu, Mete Akdoğan

**Affiliations:** ^1^Department of Electrical and Electronics Engineering, Dokuz Eylül University, Tınaztepe Campus, 35160 Izmir, Turkey; ^2^Department of Computer Engineering, Dokuz Eylül University, Tınaztepe Campus, 35160 Izmir, Turkey

## Abstract

The goal of this study was to measure the thickness of egg shells without any contact and by utilizing a nondestructive method that sends infrared light beam on the egg. We obtain measurement resolutions on the order of 7 *μ*m up to a penetration depth of 1.7 mm from the actual surface of the egg shell. The measurement results we obtained show that optical coherence tomography can be used to accurately determine the egg shell thickness. Scanning the light beam over the surface allows for measuring the egg profile and monitoring the variations of shell thickness. Since this information gives a quantitative value for the uniformity of the egg shell structure, we anticipate that optical coherence tomography may be used in the quantitative evaluation of egg quality in in-line automated inspection systems.

## 1. Introduction

The egg shell is the protective outer layer of an egg that defends the egg against damage, microbial contamination, and dehydration. Therefore, it is vital that the egg has a good quality and uniform egg shell. Many problems arise when the egg shell is thin. Some major problems are cracks that may result in the breaking of the egg, microbial contamination of the egg results in infection, and higher water vapour loss during the entire incubation process results in dehydration [1]. All these problems can cause higher embryonic mortality. For the eggs to hatch better they require therefore good quality egg shells [2]. It is then quite important to have methods that measure certain characteristics of egg shells which reveal the quality of the eggs. One method to assess egg shell quality is to measure the stiffness and damping ratio of the egg shells [3]. This technology measures the resonant frequency (RF) of the egg and its damping ratio and has proven to be a fast, objective, and nondestructive method for determining egg shell strength and is suitable for inline monitoring [4]. In a related scientific study acoustic resonance frequency analysis is used to measure egg shell quality [5].

A very recent study suggests that the egg quality is influenced not by the egg shell thickness but rather the uniformity of the egg shell thickness [6]. In this paper the scientists using a gauge measured the thickness of the egg shell along both longitudinal and latitudinal axes. The study found that the egg shell thickness from blunt to sharp end varied significantly. The egg shell was thinnest around the blunt side and thickest at the sharp egg tip.

It is clear that in order to minimize losses and maximize their efficiency the egg and poultry industry could benefit from methods that measure egg shell thickness and its variations. This then renders the quantitative measurement of the variation of egg shell thickness of eggs crucial in the quality control process. Another use of measuring egg shell thicknesses is in the investigation of the effects of pollutants and environmental contamination that causes thinning of egg shells [7]. Having accurate data of egg shell thickness will be invaluable for quantifying the hazardous effects of certain chemical wastes and pollutants on egg quality in a variety of different species. Until date there have been various different techniques employed to measure the thickness of egg shells. In general these methods are categorized into two different subgroups that are destructive and nondestructive techniques [8].

A commonly used destructive method involves drilling air holes in egg shells with micrometer screws to measure egg shell thicknesses [9]. Impact fracture force, puncture force, or quasistatic compression is other destructive methods from which the strength of the egg shell is used to infer the thickness. A standard nondestructive egg shell thickness measurement involves dipping eggs in a variety of salt solutions that have different concentrations of salt [10]. Depending on their shell thickness eggs will float in solutions with lower specific gravity. This is a simple and nondestructive method allowing for rapid testing of eggs which made the method popular. The major disadvantage of this method is that it gives crude results. Ultrasound was also shown to be a successful method to measure egg shell thickness nondestructively [11]. There are some commercial ultrasonic sensors now on the market that measure the egg shell thickness nondestructively such as (“Egg Tester Precision Ultrasonic Egg Shell Thickness Gauge,” 2013) and (“Egg shell thickness gauge,” 2013) [12, 13].

We in this paper apply the photonic imaging modality of optical coherence tomography to nondestructively measure egg shell thicknesses. We will elaborate on our experimental procedure in the next section.

## 2. Materials and Methods

We made use of an optical coherence tomography device to measure accurately egg shell thickness without touching the egg or destroying it. The method we employ is a relatively new photonic method known as optical coherence tomography (OCT). Optical coherence tomography was a method first developed in the 90s and first used in the field of ophthalmology to obtain accurate images from within the retina [14]. The working principle of the imaging technique optical coherence tomography is similar to ultrasound, simply using light waves instead of sound waves. Like in ultrasound imaging OCT utilizes the time-delay information contained in the reflected waves (photons contained in light beams for OCT) that come from different depths inside the sample. This information is then used to reconstruct detailed depth profile of the sample structure. Compared to ultrasound imaging OCT has a much better axial resolution, ~10 *μ*m for time domain—OCT and ~5 to 7 *μ*m for spectral domain—OCT versus ~150 *μ*m for ultrasound at a frequency of 10 MHz [15]. In our experiment we used a spectral domain optical coherence tomography system that allows us to measure egg shell thickness with high accuracy. Spectral domain OCT detects and separates different frequency components of the interference pattern created by the waves reflected off the sample on a charged coupled device. Different frequencies will correspond to signals coming from different depths of the sample tissue.

After taking the Fourier transform of the received signal we obtain the A-scan of the sample. B-scans are then created by assembling A-scans measured along a transverse plane on the tissue. For more information the reader may consult the nice book on Optical Coherence Tomography (Brezinski, 2006). Our OCT system had the following characteristics. The light source was an infrared super luminescent diode having a center wavelength at 930 nm and a bandwidth of 100 nm. The systems speed was A-scan line rate: 1.2 kHz, B-scan frame rate (512 lines/frame): 2 fps. The image quality was satisfied with a depth resolution of 7 *μ*m, lateral resolution of 8 *μ*m, and sensitivity of up to 105 dB. The imaging depth at this operating wavelength was 1.7 mm with a signal-to-noise ratio (SNR) of 83 dB.

**Figure 1:**
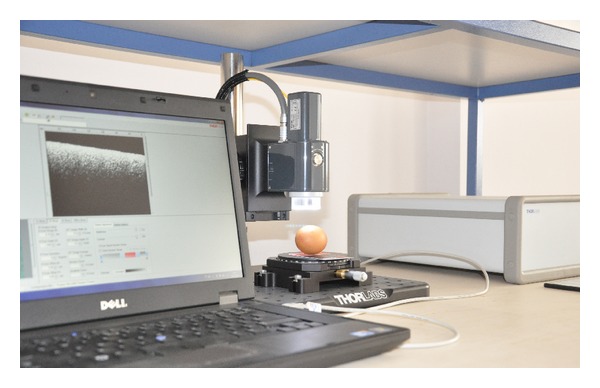
The experimental setup. The light generated by the super luminescence diode is sent through a cable and focused on the eggshell. The OCT scan can be seen on the laptops screen. The software allows for measuring desired lengths on the scan.

In the experiment, we place the egg in the sample arm. This is shown in the photo that was taken in our lab (Figure 1). The light coming from the OCT source is then focused on the surface of egg shell. This is achieved by adjusting the length of the arm and monitoring the signal reflected of the egg shell air interface. The coarse adjustment is achieved by obtaining a sharp image on the CCD camera and the fine adjustment is performed by optimizing the OCT spectrum on the B-scan. The measurement speed will be determined by the capability of the OCT system. One round trip scan around the egg requires a laser beam scan of approximately 15 cm for an average sized egg. If the measurement resolution was to be 30 *μ*m, the measurement duration would be around 4.16 seconds, taking the scan rate of our OCT (1.2 kHz for the A-scan) into account.

## 3. Results

**Figure 2:**
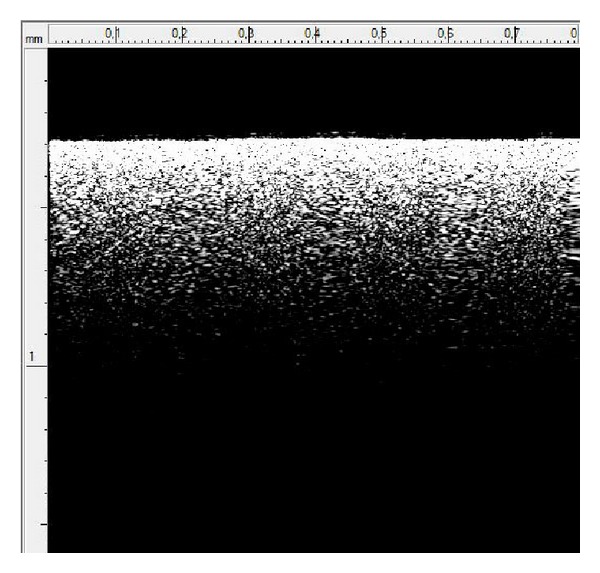
A typical OCT scan image. The brighter regions arise from the reflected light due to the refractive index mismatch between air and eggshell. The scan length is 0.8 mm and the scan depth is 1.6 mm.

In this section the results of the optical coherence tomography of the egg shells are presented.  Figure 2 shows a scan of the egg shell at a location on the egg equator. Looking from the top on the *y*-axis is the path of the light beam. The top black section then corresponds to air, and the white line is the air-egg shell interface. We can see from Figure 2 the locations in the egg shell from which there is light reflected off.

From this measurement we can directly measure the optical path length of the egg shell. For this particular point on the egg the optical path length is measured to be 560 *μ*m. This is measured between the first reflection measured on the screen and the deepest reflection one gets in the image. There is a measurement accuracy of 10 *μ*m in this measurement. It is a trivial step to estimate the actual egg shell thickness from the optical path length data by simply taking the refractive index of the shell into account. Since the egg shell is mainly comprised of calcite, its refractive index is expected to be around 1.62 at our operating wavelength. We verify this by cutting out a piece of egg shell and scanning it perpendicularly with the light beam to obtain a precise value of its thickness. The width of the shell in this scan is measured to be 340 *μ*m. Dividing the optical path length with this actual thickness measurement then gives us the effective refractive index of the egg shell of 1.63, which is in good agreement with the expected value.

The conversion of optical path length to actual egg shell thickness is not necessary when one uses the uniformity of the egg shell thickness measure. Because the coefficient of variance remains unchanged when the data set is scaled. In our experiment the optical coherence tomography of the egg shell gives us the measure of the optical path length. And this corresponds actually to the thickness of the egg shell that is scaled by the refractive index of the egg shell.

**Figure 3:**
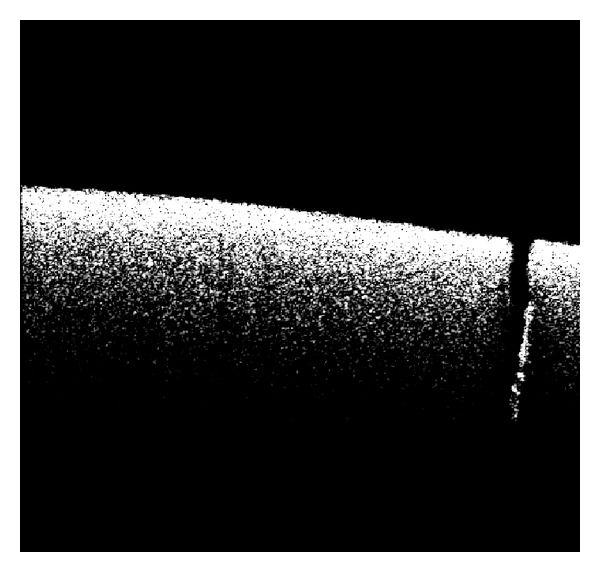
OCT crack measurement. A crack with a 30 *μ*m width is easily detected on scan. Again the software gives the exact width of the crack. This is typical OCT scan that corresponds to a height of 1.6 mm.

In Figure 3, we can see how the OCT enables detecting cracks on the egg shell surface. In the given scan we measured a crack width of 30 *μ*m.

**Figure 4:**
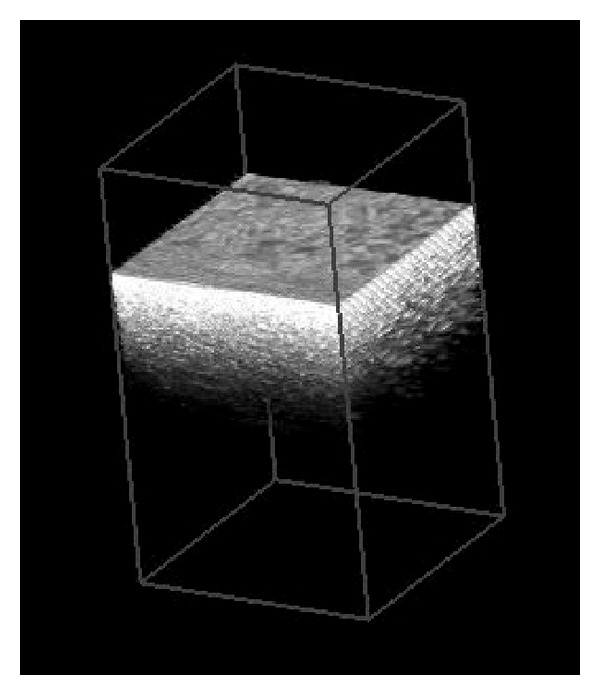
This shows a volumetric image of the egg shell. The 3D image is constructed from real OCT scans. The dimensions of the scanned volume were *x* = 0.5 mm: *y* = 0.5 mm: *z* = 1.6 mm.

This crack was visible in the OCT thanks to the high resolution (~10 um). The system also allows us to take volumetric 3-D images of the egg shell. We show the 3D scan of the egg shell in Figure 4. This image is reconstructed by scanning the light beam over a surface and combining the OCT scans to construct a 3D view.

## 4. Discussion

In this paper we successfully used an optical coherent tomography system operating at 930 nm to measure the thickness of egg shell. The OCT provides a noncontact nondestructive and micrometer high-resolution images of the egg shells from which egg shell thicknesses can be calculated with very high precision. OCT may find usage in poultry sciences in the future for nondestructive inspection of egg quality. The high resolution and accuracy of this method may render it useful in other applications in egg quality testing where minute differences or differences in egg shell characteristics in time are monitored. Our method even allows for the 3D reconstruction of the surface of the measured egg. A further study will be to use these volumetric high resolution image data to measure egg quality quantitatively. OCT due to its very high resolution may prove useful in also measuring cracks in the egg shells and analyzing their structure. This method upon further investigation may prove to be complimentary to the already existing crack detection methods such as candle light inspection, acoustic resonance frequency analysis [16], and pattern recognition algorithms [17]. Recently a group of scientists have been conducting research on the thickness variation of dinosaur eggs. Optical coherence tomography could be considered as a method of investigating the structure of different dinosaur egg shells [18]. Our imaging system allows for a very rapid and accurate measurement of the egg shell thickness uniformity parameter defined for the quality of eggs. Moreover this is the first study up to our knowledge that used optical coherence tomography in order to detect egg shell cracks. Therefore we see the potential of the usage of our system in automated in-line quality control monitoring of eggs in the field of poultry.
